# Comparing snowball sampling and RDS: A methodology and case study

**DOI:** 10.1371/journal.pone.0331666

**Published:** 2026-01-14

**Authors:** Dongah Kim, Krista J. Gile, Bradley Mathers, Massimo Mirandola, Lorenzo Gios, Igor Toskin, Keith M. Sabin

**Affiliations:** 1 Integrative Biology Department, University of Texas Austin, Austin, Texas, United States of America; 2 Mathematics and Statistics Departments, University of Massachusetts Amherst, Amherst, Massachusetts, United States of America; 3 UNDP-UNFPA-UNICEF-WHO-World Bank Special Programme of Research, Development and Research Training in Human Reproduction (HRP), Department of Sexual and Reproductive Health and Research, World Health Organization, Geneva, Switzerland; 4 Infectious Diseases Section, Department of Diagnostics and Public Health, University of Verona, Verona, Italy; 5 UNAIDS, Geneva, Switzerland; John Paul II Catholic University of Lublin: Katolicki Uniwersytet Lubelski Jana Pawla II, POLAND

## Abstract

Both snowball sampling and Respondent Driven Sampling (RDS) are used to sample hard-to-reach populations. Snowball sampling was initially developed as a probability sampling method, but in practice, it is widely used as a non-probabilistic sampling method. RDS was developed to address the limitations of snowball sampling and can be used to approximate a probability sampling method in practice. Therefore, RDS is often recommended for bio-behavioral surveys (BBS) for surveillance of HIV, viral hepatitis, and STIs among key populations. In some settings, simpler and cheaper monitoring are desired. WHO and UNAIDS are developing a simplified and rapid bio-behavioral survey methodology, a version of snowball sampling to use when RDS is infeasible. In this paper, we use data-based simulations to examine the potential similarities and differences between results from a snowball sample with recruitment initiated from a health service and samples recruited through RDS methodology.

## Introduction

Both snowball sampling [1,2] and Respondent Driven Sampling (RDS) [3–5] are commonly used to collect samples from populations where standard sampling approaches are not appropriate or prohibitively expensive, but the population is well-connected by a social network. Both sampling methods start with a small initial sample, which is expanded by recruiting from within the social networks of previous participants. Many statistical methods have been developed for obtaining valid estimates from data obtained from snowball sampling when the sample begins with a probability sample [1,6–8]. However, getting an initial sample through random sampling is usually challenging. The reliance on an initial convenience sample violates this condition and renders the whole sample a non-probability sample. The dependence on the initial sample is further heightened when there are large numbers of initial samples and few steps or waves of sampling away from the initial sample. In practice, snowball samples are typically treated as convenience samples. Non-network-based sampling methods such as time-location [9,10], venue-based [11], and targeted sampling [12] also require strong assumptions for valid inference for hard-to-reach populations. RDS has several innovations to address the limitations of snowball sampling. First, RDS limits the number of initial seeds to be small and only allows for a limited number of recruits per respondent, which results in longer sample chains (more waves of recruitment) for a desired sample size. This reduces the dependence of the final sample on the initial seeds. Second, in the presence of key assumptions, an RDS sample can be treated as a probability sample for statistical inference [3,13–15]. Third, to further aid practicality in stigmatized populations, RDS also allows for anonymous recruitment. The sample expands when respondents distribute a small number of uniquely identified coupons among their contacts, making them eligible for participation. Because these innovations allow for practical sampling and more valid statistical inference, RDS is used in many fields and many countries, especially for surveillance of high-risk hard-to-reach populations [16–20].

Bio-behavioral surveys (BBS) [21] for surveillance of HIV, viral hepatitis, and sexually transmitted infections among key populations (such as men who have sex with men, sex workers, and people who inject drugs) commonly use RDS to recruit these groups which are typically hard to reach through other sampling methods. BBS that employs these methods typically requires substantial financial and technical resources. WHO and UNAIDS are developing a simplified and rapid survey methodology that is intended to be less expensive, less technically demanding than a BBS, and able to be implemented on a regular basis by providers of HIV and other health services to these populations. This ‘BBS-Lite’ methodology involves the consecutive sampling of eligible clients accessing health services, who are then provided coupons to recruit other participants through snowball sampling, with an anticipated limited number of waves. To evaluate the strengths and limitations of this proposed methodology, we undertook a simulation study using existing data to compare RDS and snowball sampling methods.

## Methods

This paper presents a simulation-based framework to compare two possible sampling methods. In particular, we compare a specific version of snowball sampling modeled after the method planned by WHO and UNAIDS (here called BBS-Lite snowball sampling) and RDS to find possible biases in BBS-Lite as compared to RDS. To best approximate the types of covariate dependence we might be likely to see in real sampling, we used data from previous surveys using RDS in key populations of interest as the basis for our simulation study. This simulation method can be used with existing RDS data to assess whether a BBS-Lite style study is advisable in a given population. A key limitation of this method is that RDS samples have a tree-structure, while full populations are connected by more complex network structures. In most cases, however, RDS samples provide the best, or only, information about the dependence patterns in populations sampled by link-tracing network samples. To keep our study as close to these data as possible, we focus on the outcome that is most observable: comparing sample composition between RDS and BBS-Lite Snowball Sampling. In particular, we focus on the implications of two critical differences between the two methods: the selection of the initial sample and the depth of sampling.

We simulate both BBS-Lite snowball sampling and RDS using data collected in the Sialon II bio-behavioral study [22], which used RDS to recruit men who have sex with men across several European populations. Then, we compare the original data and the simulated data to examine the potential similarities and differences between results from a BBS-Lite snowball sample and a same-sized RDS sample.

### Introduction to the data

**Fig 1:**
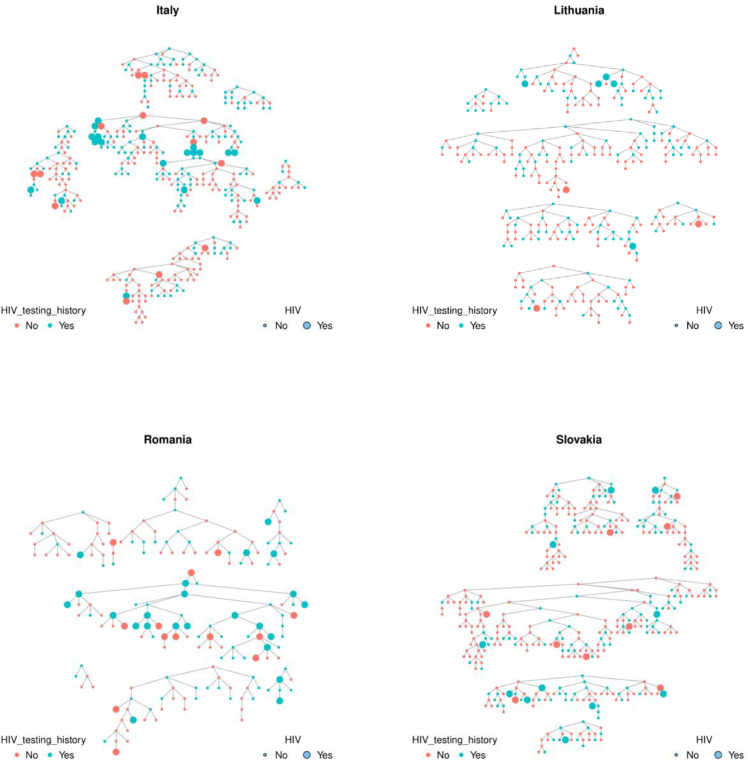
Sialon II data from RDS. Colors represent the health service users status (here, HIV testing history) and circle sizes indicate HIV status.

The Sialon II project [22] is a multi-center biological and behavioral cross-sectional survey carried out across European countries using Time-Location Sampling (TLS) and RDS between 2013 and 2014 to better understand the HIV/STI prevention needs and prevention regulation gaps of Men who have Sex with Men (MSM). RDS was used to recruit a total of 1,305 participants in four countries (400 from Italy, 322 from Lithuania, 183 from Romania, and 400 from Slovakia); see Fig 1 and Table 1. The number of initial sample seeds ranged from 5 to 9, and the maximum number of waves of recruitment in each study ranged between 8 and 21.

**Table 1:** Sialon II Data description.

	n	seed	mean.wave	max.wave	Receive Condoms(%)	HIV testing history (%)
Italy	400	6	7.53	21	141 (0.3525)	178 (0.4450)
Lithuania	322	6	4.47	11	162 (0.5031)	129 (0.4006)
Romania	183	9	4.01	8	105 (0.5738)	87 (0.4754)
Slovakia	400	5	6.58	15	103 (0.2575)	149 (0.3725)

Because the BBS-Lite snowball method begins with an initial sample of health service users, we identified questionnaire items from the Sialon II survey that would indicate a participant was a health service user. The following two variables were used as proxy indicators of health service utilization: 1) “Have you been given condoms at drop-in centers, sexual-health clinics, health care facilities, outreach service/gay/HIV/other association in last 12 months?” (referred to here as ‘Receive condoms’); and 2) “Have you been tested for HIV in the last 12 months?” (referred to here as ‘HIV testing history’). We summarize the number of health service users according to these variables in Table 1. These two variables were selected as proxies for health service utilization because condom distribution and HIV testing typically take place at locations that provide HIV-related care services.

We then examined the sample means and estimated population proportions using the RDS II estimates [14] for several variables we selected for comparison. Variable names and abbreviations are in Table 2, sample means are in Table 3 and RDS II estimates are in Table 4.

**Table 2:** Variable names and abbreviations.

Abbreviation	Variable name
Age	Age
HIV	What was the result of your last HIV test?
Receive condoms	Have you been given condoms at drop-in centers, sexual-health clinics, health care facilities, outreach service/gay/HIV/other association in last 12 months
HIV testing History	Have you been tested for HIV in the last 12 months?
Other testing history	In the last 12 months, have you been tested for sexually transmitted infections other than HIV?
ART coverage	In case you are living with HIV: are you currently taking drugs for treatment for HIV (known as antiretroviral, ART, HAART)?
NSMP	In the last 6 months, how many male non-steady partners have you had sex with?
Unprotected NSMP	In the last 6 months, how many male non-steady partners have you had unprotected (without condom) anal intercourse with?
Homosexuality	How you think of yourself? Gay or Homosexual?
Injected Drug	Have you ever injected drugs?
Employed status	Are you current employed (full-time or part-time)?

**Table 3:** Unadjusted sample means, Sialon II data.

	Age	HIV	Receive Condoms	HIV testing History	Other testing History	ART Coverage
Italy	32.12	0.0800	0.3525	0.4450	0.3875	0.5313
Lithuania	30.22	0.0248	0.5031	0.4006	0.2889	0.2500
Romania	30.26	0.1967	0.5738	0.4754	0.3770	0.0833
Slovakia	29.49	0.0500	0.2575	0.3725	0.2950	0.1000
	**NSMP**	**Unprotected NSMP**	**Homosexuality**	**Injected Drug**	**Employed Status**	
Italy	5.9775	1.2350	0.6692	0.0150	0.4677	
Lithuania	3.8230	0.7422	0.6978	0.0341	0.6947	
Romania	4.7049	1.0273	0.4056	0.2022	0.4176	
Slovakia	4.4600	0.9250	0.7261	0.0125	0.5581	

**Table 4:** Sample mean estimates using RDS II estimates.

	Age	HIV	Receive Condoms	HIV testing History	Other testing History	ART Coverage
Italy	31.93	0.0966	0.3456	0.4109	0.3493	0.4357
Lithuania	30.70	0.0354	0.3900	0.3300	0.2523	0.1613
Romania	30.83	0.1802	0.5128	0.4555	0.3565	0.0551
Slovakia	30.32	0.0421	0.2177	0.3405	0.2754	0.1256
	**NSMP**	**Unprotected NSMP**	**Homosexuality**	**Injected Drug**	**Employed Status**	
Italy	5.4761	1.8088	0.5827	0.0209	0.4288	
Lithuania	2.8805	0.6036	0.6281	0.0354	0.6804	
Romania	3.9390	0.5983	0.3698	0.1782	0.4622	
Slovakia	3.6872	0.8969	0.7013	0.0112	0.5825	

### Data generating process

To match our knowledge of the true network as closely as possible, we simulated samples with replacement directly from the sampled network trees of the original RDS data. This process guarantees that each adjacent sampled person is indeed adjacent to their simulated recruiter. It also preserves the observed rates of mixing across subgroups in the population. We simulated samples both using BBS-Lite Snowball Sampling, and RDS, with the latter serving as a check for artifacts of the sampling process induced by our re-sampling procedure over trees rather than a full network. Our primary interest is in whether the simulated samples approximate the original RDS samples.

This study focuses on differences between RDS and BBS-Lite Snowball Sampling resulting from two key differences between the two methods: the composition of the initial sample and the depth or the number of waves of sampling. For our simulation of snowball sampling, we selected initial seeds from the participants who responded “yes” to our service use question (in separate simulations, this is either the ‘Receive condoms’ or ‘HIV testing history’ variable). For RDS, seeds were chosen at random. In RDS, the number of initial seeds and the number of people that one person can recruit are small (usually 2-3 recruits in practice), and there is no limit on the number of waves. For our simulation of BBS-Lite snowball sampling, we limited the number of waves to a maximum of 2 and the potential number of participants recruited from an individual to 3 or fewer. With these constraints, for the simulated BBS-lite snowball sample to attain the same sample size as RDS, the number of initial seeds (i.e., those recruited through HIV services) was considerably larger. We summarize these 3 sampling conditions in Table 5.

**Table 5:** Several sampling conditions with different seed characteristics.

	Seed Characteristic	number of seeds	number of Waves	number of coupons
Snowball Sampling	Receive condoms	no restriction	2	3
	HIV testing history	no restriction	2	3
RDS	chosen at random	matching original data	no restriction	3

The detailed sampling steps were:

- BBS-Lite/Snowball
  1. Randomly select one initial seed from the service-users group (according to ‘Receive condoms’ or ‘HIV testing history,’ depending on the simulation setting).
  2. Randomly assign 0-3 as the number of recruits.
  3. Sample with-replacement the assigned number of recruits from among respondents linked to the recruiter in the original RDS data. (Add the new recruits to the set of potential new recruiters.)
  4. Repeat steps 2-3 once to sample 2 waves.
  5. Repeat steps 1-4 until the desired sample size is reached.
- RDS
  1. Randomly select seeds from the original data. The number of seeds is the same as the original data.
  2. Select the first node in the sample that has not yet served as recruiter.
  3. Randomly assign 0-3 as the number of recruits for this recruiter.
  4. Sample with-replacement the assigned number of recruits linked to the recruiter in the original RDS data.
  5. Repeat steps 2-4 until desired sample size is reached.

Each sampling procedure was repeated 1000 times.

### Measures related to sample differences

We focused on two population structures, reflected in our data and in other RDS studies, that might induce bias in BBS-Lite samples, as compared to RDS: the complete inaccessibility of some people in BBS-Lite, and the dependence between the service variable used to seed the RDS study and variables of interest.

The structure of BBS-Lite snowball sampling means that only population members within 2 network steps of health service users can be sampled; the rest of the population is inaccessible. This is true in a real population and also in our simulations. To study the impact of this non-accessibility, we compared the sample composition of accessible and inaccessible respondents in the Sialon II data. The results are in Table 6, which includes the results of nominal $\chi^{2}$-tests comparing the accessible and inaccessible groups for each variable of interest. As expected, the service usage variables used to select seeds differed dramatically across the accessible and inaccessible groups in all cases. Many other variables had nominally significant differences between the accessible and inaccessible groups. In these cases, we may expect to see important biases in BBS-Lite compared to RDS.

**Table 6:** Sample composition of accessible and inaccessible samples based on seed characteristics.

Seed	Receive Condoms							
	Italy		Lithuania		Romania		Slovakia	
Accessible	Yes	No	Yes	No	Yes	No	Yes	No
N	360	40	310	12	180	3	308	92
Sample proportion	90%	10%	96.23%	3.73%	98.36%	1.64%	77%	23%
Age (mean)	32.36	29.85	30.13	32.58	30.33	26	29.57	29.21
HIV	0.0833	0.05	0.0226	0.0833	0.2000 ^***^	0	0.0422	0.0761
Receive Condoms	0.3917 ^***^	0	0.5226 ^***^	0	0.5833 ^***^	0	0.3344 ^***^	0
HIV testing history	0.4778 ^***^	0.15	0.4097	0.1667	0.4778	0.3333	0.3799	0.3478
Other thesting history	0.4139 ^***^	0.15	0.2968	0.0833	0.3778	0.3333	0.2987	0.2826
ART Coverage	0.5667 ^***^	0	0.2857	0	0.0833	0	0.1538	0
NSMP	6.2833 ^**^	3.2250	3.7613	5.4167	4.7111	4.333	4.0130	5.9565
Unprotected NSMP	1.3389 ^**^	0.3	0.7290	1.0833	1.0333	0.6667	0.8734	1.0978
Homosexuality	0.6886	0.5	0.6990	0.6667	0.4011	0.6667	0.7255	0.7283
Injected Drug	0.0167	0	0.0355 ^***^	0	0.2056 ^***^	0	0.0162	0
Employed Status	0.4582	0.5500	0.6958	0.6667	0.4190	0.3333	0.5428	0.6087
**Seed**	**HIV testing history**							
	**Italy**		**Lithuania**		**Romania**		**Slovakia**	
Accessible	Yes	No	Yes	No	Yes	No	Yes	No
N	374	26	294	28	176	7	368	32
Sample proportion	94.5%	6.5%	91.3%	8.7%	96.17%	3.83%	92%	8%
	26 (6.5%)	294 (91.3%)	28 (8.7%)	176 (96.17%)	7 (3.83%)	368 (92%)	32 (8%)	
Age (mean)	32.04	33.28	30.28	29.57	30.25	30.43	29.60	28.22
HIV	0.0802	0.0769	0.0238	0.0357	0.1989	0.1429	0.0516	0.0313
Receive Condoms	0.3556	0.3077	0.5170	0.3571	0.5852	0.2857	0.2554	0.2813
HIV testing history	0.4759 ^***^	0	0.4388 ^***^	0	0.4943 ^***^	0	0.4049 ^***^	0
Other testing history	0.4091 ^***^	0.0769	0.3129 ^***^	0.0357	0.3920 ^***^	0	0.3152 ^***^	0.0625
ART Coverage	0.5667 ^***^	0	0.2857	0	0.0857	0	0.1053	0
NSMP	6.2219 ^***^	2.4615	3.9354	2.6429	4.1591	1.8428	4,4837	4.1875
Unprotected NSMP	1.3192 ^**^	0.1538	0.7279	0.8930	1.2084	1	0.9484	0.6562
Homosexuality	0.6703	0.6538	0.7065	0.6071	0.3931	0.7143	0.7295	0.6875
Injected Drug	0.0160	0	0.034	0.0357	0.2102 ^***^	0	0.0136	0
Employed Status	0.4475 ^**^	0.7600	0.7031	0.6071	0.4229	0.2857	0.5522	0.6250

tests of association,  p < 0.05, ^**^p < 0.01, ^***^ p < 0.001.

An association between the seed and target variables may also induce bias (summarize in Table 7). To measure this, we used a semiparametric test for bivariate association (SPRTBA) [23] designed to infer binary relationships between categorical data in RDS samples, for categorical variables and logistic regression as a heuristic for continuous variables (‘Age,’ ‘NSMP’ and ‘Unprotected NSMP’). Because these two tests used different test statistics, we report only the p-values of each test. Note that the logistic regression may have inflated the type-I error rates due to the dependence on the RDS sample, so we interpret this Table 7 as a heuristic guide rather than a formal statistical test.

**Table 7:** SPRTBA for categorical variables or logistic regression for continuous variables (‘Age’, ‘NSMP’ and ‘Unprotected NSMP’) p-values between seed variables and the selected variables by country.

Seed	Receive Condoms			
	Italy	Lithuania	Romania	Slovakia
Age	0.3139	0.0000 †††	0.1103	0.1568
HIV	0.8072	0.9230	0.0040 ††	0.6593
Receive Condoms	0.0000 †††	0.0000 †††	0.0000 †††	0.0000 †††
HIV testing History	0.0000 †††	0.0000 †††	0.0000 †††	0.0270 †
Other testing History	0.0000 †††	0.0000 †††	0.0070 ††	0.0060 ††
ART Coverage	0.1978	0.2226	0.2018	0.9039
NSMP	0.0001 †††	0.0504	0.5860	0.1780
Unprotected NSMP	0.0440 †	0.4250	0.7251	0.6740
Homosexuality	0.2268	0.0000 †††	0.8162	0.4436
Injected Drug	0.3862	0.3926	0.0120 †	0.8780
Employed Status	0.3646	0.1139	0.0909	0.6094
**Seed**	**HIV testing history**			
	**Italy**	**Lithuania**	**Romania**	**Slovakia**
Age	0.0715	0.0059††	0.9180	0.4013
HIV	0.1139	0.2128	0.0400 †	0.1009
Receive Condoms	0.0000 †††	0.0000 †††	0.0000 †††	0.0220 †
HIV testing History	0.0000 †††	0.0000 †††	0.0000 †††	0.0000 †††
Other testing History	0.0000 †††	0.0000 †††	0.0000 †††	0.0000 †††
ART Coverage	0.0050 ††	0.0949	0.0873	0.2742
NSMP	0.0038††	0.0366 †	0.2880	0.0265 †
Unprotected NSMP	0.8860	0.8982	0.8060	0.0699
Homosexuality	0.0030 ††	0.0240 †	0.2887	0.2927
Injected Drug	0.2783	0.8290	0.0000 †††	0.3011
Employed Status	1.0000	0.5135	0.3217	0.2627

tests of association, † p < 0.05, †† p < 0.01, ††† p < 0.001.

### Bias measurement

We evaluated the performance of each simulation setting by comparing the composition of the set of the simulated samples to the composition of the original true RDS data. If the sampling method does not impact sample composition, we expect the observed data composition to be “typical” of the simulated samples. To measure this, we used the quantiles of the observed sample mean among the sample means of each simulated dataset using a measure we called *d*_*Q*_:

$$d_{Q}=2\times |\frac{1}{T}\sum_{i=1}^{T}1(X_{i}\leq x_{obs})-0.5|.$$ (1)

Here, *X*_*i*_ are simulated samples, *x*_*obs*_ is the value from the original RDS data, and 𝟙 is an indicator function taking the value 1 when its argument is true, and 0 otherwise.

## Results

**Fig 2:**
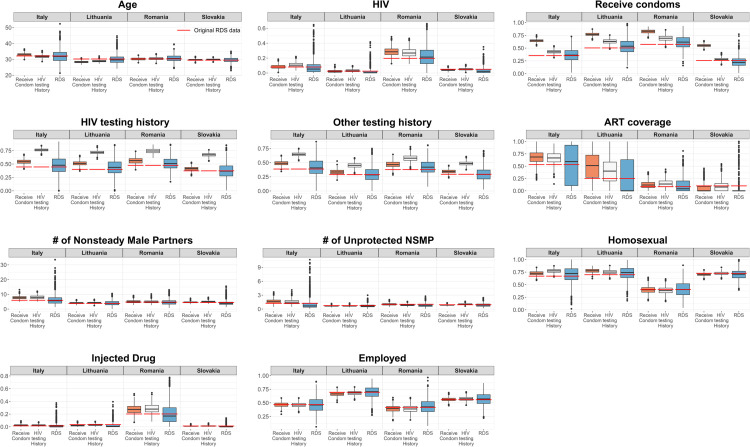
Sample composition with 3 resampling methods: BBS-Lite snowball sampling with two possible seed settings, and RDS resampling.

The primary study results are summarized in Fig 2. Each set of 3 boxplots compares the 1000 simulated samples in each of the 3 simulated sampling conditions with the sample mean of the original RDS data. Boxplots where the red line is far from the middle indicate simulations with biased sample compositions compared to the original RDS data.

Table 8 gives the corresponding measures of *d*_*Q*_. This Table 8 also indicates the nominal significance levels of the tests comparing accessible and inaccessible samples, as well as the tests for association with seed variables. We see that in nearly all cases when there are nominally significant differences between accessible and inaccessible samples, there is substantial bias in BBS-Lite Snowball Samples. However, this does not explain all observed biases (for example, Age variable in Lithuania (Fig 2)). In almost all the remaining cases of substantial bias, the association test is nominally significant.

**Table 8:** The measure of *d*_*Q*_. The  indicates a significant difference between the accessible and inaccessible samples through the $\chi^{2}$-test. The † indicates a significant correlation between Seed variables and selected variables.

Variable	Country	BBS-Lite Snowball Sampling		RDS
		Receive Condom	HIV testing History	
Age	Italy	0.271	0.162	0.034
	Lithuania	0.495† ††	0.470††	0.099
	Romania	0.013	0.090	0.051
	Slovakia	0.138	0.266	0.012
HIV	Italy	0.009	0.291	0.147
	Lithuania	0.152	0.076	0.269
	Romania	0.442^***^††	0.377†	0.034
	Slovakia	0.273	0.046	0.253
Receive Condoms	Italy	0.500^***^†††	0.484†††	0.037
	Lithuania	0.500^***^†††	0.496†††	0.081
	Romania	0.500^***^†††	0.477†††	0.121
	Slovakia	0.500^***^†††	0.223†	0.144
HIV Testing History	Italy	0.492^***^†††	0.500^***^†††	0.072
	Lithuania	0.498†††	0.500^***^†††	0.082
	Romania	0.409†††	0.500^***^†††	0.089
	Slovakia	0.350†	0.500^***^†††	0.015
Other Testing History	Italy	0.495^***^†††	0.500^***^†††	0.049
	Lithuania	0.330*† ††	0.500^***^†††	0.019
	Romania	0.410††	0.500^***^†††	0.101
	Slovakia	0.406††	0.500^***^†††	0.007
ART Coverage	Italy	0.368^***^	0.345^***^††	0.037
	Lithuania	0.253	0.188	0.190
	Romania	0.165	0.265	0.087
	Slovakia	0.291	0.097	0.397
NSMP	Italy	0.460^**^†††	0.469^***^††	0.048
	Lithuania	0.155†	0.205†	0.026
	Romania	0.116	0.095	0.078
	Slovakia	0.116	0.290†	0.186
Unprotected NSMP	Italy	0.246^**^††	0.170^**^	0.238
	Lithuania	0.231	0.254	0.148
	Romania	0.035	0.245	0.219
	Slovakia	0.185	0.284	0.091
Homosexuality	Italy	0.404	0.495††	0.122
	Lithuania	0.473†††	0.391†	0.123
	Romania	0.038	0.134	0.010
	Slovakia	0.252	0.021	0.096
Injected Drug	Italy	0.148	0.165	0.208
	Lithuania	0.329^***^	0.148	0.261
	Romania	0.342^***^†	0.365^***^†††	0.083
	Slovakia	0.152	0.081	0.276
Employed Status	Italy	0.009	0.061*	0.024
	Lithuania	0.256	0.140	0.036
	Romania	0.164	0.164	0.032
	Slovakia	0.049	0.181	0.043

tests of association,  p < 0.05, ^**^p < 0.01, ^***^ p < 0.001 tests of association, † p < 0.05, †† p < 0.01, ††† p < 0.001.

**Fig 3:**
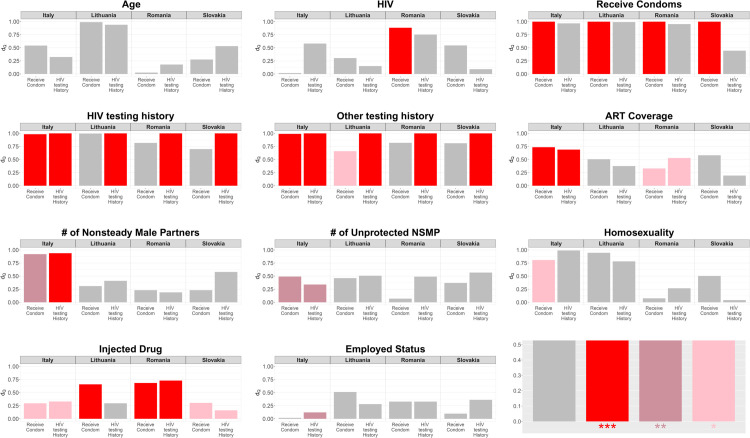
Difference between simulated samples and true data ($d_{Q}$) as related to significance level of difference between the accessible and inaccessible samples through the $\chi^{2}$-test. The bar height represents the measure of *d*_*Q*_, and the color represents the significance levels of the $\chi^{2}$-test. The last plot shows the significance levels.  indicates a significant difference between the accessible and inaccessible samples through the tests of association ( p-value < 0.5, ^**^p-value < 0.1, ^***^p-value < 0.01).

**Fig 4:**
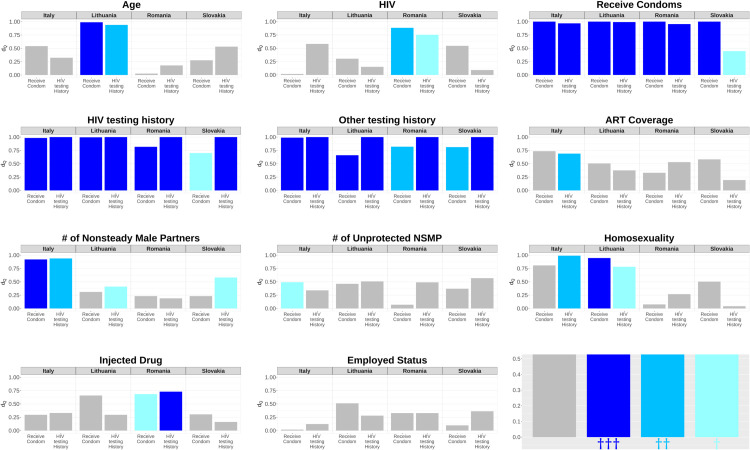
Difference between simulated samples and true data ($d_{Q}$) as related to significance level of SPRTBA/logistic regression for finding an association between seed variables and selected variables. The bar height represents the measure of *d*_*Q*_, and the color represents the significance levels of the SPRTBA/logistic regression. The last plot shows the significance levels. † indicates a significant association using SPRTBA/logistic regression between Seed variables and selected variables (^†^p-value < 0.5, ^††^p-value < 0.1, ^†††^p-value < 0.01).

Figs 3 and 4 plot *d*_*Q*_ colored by the nominal significance levels. In Fig 3, we see that the high bars (*d*_*Q*_ large) are often, but not always colored, and several low bars are colored, suggesting that the difference between the accessible and inaccessible populations is a helpful but not wholly reliable indicator of bias. In Fig 4, almost all the high bars are colored, and the low bars are not. This suggests that the association between the seed and target variables is a more reliable indicator of bias. We also note that when there is a nominally significant difference between accessible and inaccessible samples, there is also a nominally significant association in most cases.

**Fig 5:**
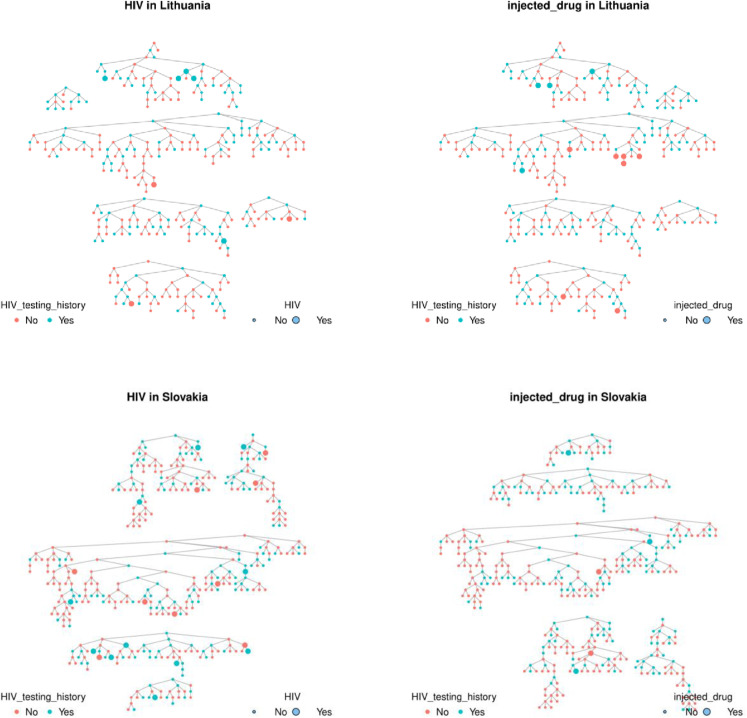
Sialon II data from Lithuania and Slovakia by ‘HIV’ and ‘Injected Drugs’.

We also consider the simulated RDS sampling results, as a sanity check. If the simulated RDS samples differ systematically from the original RDS data, then the biases we are seeing in the simulated BBS-Lite Snowball Samples may be due to features other than the approximations of the BBS-Lite structure. In Table 8, we see that for RDS re-sampling, most *d*_*Q*_ values are small. However, we find that the variables ‘HIV’, ‘Unprotected NSMP’, ‘ART Coverage’ and ‘Injected Drug’ are quite high in some countries (0.2<*d*_*Q*_<0.3). In the cases of the ‘HIV’ and ‘Injected Drug’ variables, the sample proportion is very small. In particular, in Lithuania and Slovakia, the sample proportion of ‘HIV’ and ‘Injected Drug’ is less than 0.05. We see from Fig 5, there are very few cases of HIV positive or injecting drug user respondents, so when sampling with a small number of seeds, HIV positive or injecting drug user respondents are less likely to be sampled. Because of the small numbers in these groups, the probability of sampling someone who is HIV positive or injected drugs depends strongly on the specific selection of seeds. In the case of the ‘Unprotected NSMP’ and ‘ART Coverage’ variables, the number of informative samples responding to these variables is small. ‘ART Coverage’ applies only to HIV-positive cases, and ‘Unprotected NSMP’ also has few non-zero respondents. Therefore, the resamples have high variance, and bias in RDS resamples, as compared to the RDS reference, occurs largely in the cases of very small sample fractions.

## Discussions

In this project, we introduced a method for studying the implications of different network-based sampling methods on sample composition. In particular, we consider the implications of snowball sampling as intended under BBS-Lite, including a restricted number of waves and selecting only seeds accessing health services, as compared to standard BBS sampling using RDS. The method uses previously-sampled RDS data, which may be available in settings considering BBS-Lite sampling.

The simulation found that the sample compositions of RDS re-samples were largely consistent with the original data, while the sample compositions from the BBS-Lite snowball re-samples were often quite different from the original data. We measured these differences using *d*_*Q*_, a measure reflective of bias scaled by variability.

We compare these methods on 11 target variables in data from 4 countries, with a range of variable and sample characteristics. We also consider 2 diagnostic measures, which can be computed based on RDS data alone and are associated with features we expect to be related to bias induced by the limitations of BBS-Lite Snowball Sampling. We find that nominal tests of association between the seed variable and the variables of interest are reliable indicators of substantial differences in sample composition between RDS and BBS-Lite Snowball Sampling. We expect that this is because such dependence induces over (or under) representation of the variable of interest in the BBS-Lite snowball samples with their short sample chains beginning with a given seed population. The bias is exacerbated by homophily on the variable of interest, which induces strong dependence between the initial sample and all other samples collected within the 2 waves in the BBS-Lite approach.

We also consider differences in the sample composition of accessible and inaccessible subsets of nodes based on the BBS-Lite sampling strategy. In the simulated BBS-Lite Snowball Sampling setting, we only recruit two steps away from the initial seeds, making some parts of the original data (and of real populations) inaccessible to the snowball samples. We find that if the accessible and inaccessible groups are nominally significantly different with respect to a target variable, the BBS-Lite sample composition is usually also biased with respect to the RDS sample composition, although this result is not as consistent as the association diagnostic, and the absence of nominal difference in accessibility does not assure similar sample composition.

We found a few cases where the sample proportion of both BBS-Lite and RDS re-samples were biased or had extremely high variance. These instances corresponded to variables with very little variability in the original data. It is also of note that the variance of estimates tends to be higher with RDS re-sampling than with snowball re-sampling. This is because of the greater mixing of the RDS samples which also increases the representativeness of those samples.

Our study here has focused on the difference in sample composition between RDS and BBS-Lite. This is because the assumptions needed for inference from RDS data are clearly not met by BBS-Lite. This means the BBS-Lite samples should not be used to directly estimate population proportions. We hope that our study has shown some conditions when there should be greater or lesser comparability between a BBS-Lite sample and an RDS sample. In cases where we have some confidence the samples may be comparable, BBS-Lite studies executed between less frequent RDS studies might be used to monitor population change compared to benchmark RDS data.
